# Platelet Activation Is Not Always Associated With Platelet-Related Plasma microRNA Abundance – Results From a Randomized Controlled Trial of Periodontal Patients

**DOI:** 10.3389/fphys.2021.613515

**Published:** 2021-03-01

**Authors:** Stefan Heber, Markus Laky, Isabella Anscheringer, Lukas Wolschner, Marion Mussbacher, Teresa Krammer, Hady Haririan, Waltraud C. Schrottmaier, Ivo Volf, Matthias Hackl, Andreas Moritz, Alice Assinger

**Affiliations:** ^1^Center for Physiology and Pharmacology, Institute of Physiology, Medical University of Vienna, Vienna, Austria; ^2^Division of Conservative Dentistry and Periodontology, School of Dentistry, Medical University of Vienna, Vienna, Austria; ^3^Center for Physiology and Pharmacology, Institute of Vascular Biology and Thrombosis Research, Medical University of Vienna, Vienna, Austria; ^4^Department of Pharmacology and Toxicology, University of Graz, Graz, Austria; ^5^TAmiRNA GmbH, Vienna, Austria

**Keywords:** miRNAs, biomarkers, P-selectin, atherosclerosis, platelet

## Abstract

Platelets are involved in a variety of diseases, making their adequate functional assessment is essential. However, due to their easily activatable nature this has some methodological pitfalls. Therefore, the availability of stable, easily measurable surrogate markers would be beneficial. In this regard, some evidence suggests that certain microRNAs (miRNAs) circulating in plasma might be useful. We aimed to corroborate their suitability by analyzing plasma samples obtained in a randomized controlled trial, which assessed the effects of periodontal treatment on platelet function. We hypothesized that miRNA levels mirror changes of platelet activation and -function. Both platelet function and miRNA abundance were quantified using state-of-the-art flow cytometry and qPCR methods. The following miRNAs were quantified: 223-3p, 150-5p, 197-3p, 23a-3p, 126-3p, 24-3p, 21-5p, 27b-3p, 33a-5p, 320a, 191-5p, 28-3p, 451a, 29b-3p, and 1-3p. However, periodontal treatment did not affect the abundance of any investigated miRNAs to a relevant extent. Platelet activation and reactivity indices did neither correlate with any tested miRNA at baseline, nor after the treatment period. In addition, there was no evidence that investigated miRNAs were released by platelets, as suggested previously. In conclusion, our data suggest that in patients suffering from periodontal disease the investigated miRNAs are unlikely to be suitable biomarkers for platelet function. Our data aim to raise awareness that previously determined platelet activation dependent circulating miRNAs are not suitable as platelet biomarkers in all cohorts.

## Introduction

Platelets play a crucial role in hemostasis (Semple et al., 2011), immune responses (Kral et al., 2016), host defense (Yeaman and Bayer, 2013), and atherogenesis (Lievens and von Hundelshausen, 2011). Increased platelet activation and hyperreactive platelets have been linked to atherogenesis both in experimental animal studies (Huo et al., 2003) as well as in observational clinical studies (Blake et al., 2003). Thus, it is important to tightly monitor platelet function, as it predicts cardiovascular events such as myocardial infarctions (Puurunen et al., 2018).

Platelet function analysis however has some pitfalls, which limits implementation of platelet function tests in clinical routine, e.g., due to spontaneous activation (Beaulieu and Freedman, 2013), which makes immediate function testing mandatory. Therefore, more stable surrogate markers for platelet activation, e.g., in plasma, could provide an attractive alternative. In this context, circulating microRNAs (miRNAs) could be of interest as some have recently been shown to correlate with platelet activation and function (Nagalla et al., 2011; Sunderland et al., 2017) and thus might be used as biomarkers (Willeit et al., 2013). Platelets reportedly inherit miRNAs from megakaryocytes (Opalinska et al., 2010), but also take up RNAs from the environment (Kirschbaum et al., 2015) and release these miRNAs upon activation. In line with this, treatment with anti-platelet drugs reduced the abundance of several miRNAs in plasma (Willeit et al., 2013). In turn, some miRNAs have also been described to have an impact on platelet function. For instance, miR-126 was found to affect platelet adhesion, degranulation, and thromboxane A2-generation (Kaudewitz et al., 2016). Given this body of evidence, an association between platelet function and the amount of certain circulating miRNA seems plausible. Moreover, miRNAs can be determined in previously frozen and thus storable plasma samples. Therefore, we analyzed the robustness of miRNAs as markers of platelet function.

The aim of the present study was to corroborate the applicability of certain miRNAs determined in plasma as biomarkers for platelet activation and function. We chose a panel of microRNAs that were previously described to be associated with platelet function and quantified miRNAs in plasma samples of patients with periodontal disease from a randomized controlled trial (Laky et al., 2018). In this trial intensive periodontal treatment prevented exacerbation of platelet activation. Based on that, we hypothesized (i) that the treatment also affects the abundance of certain miRNAs in plasma and (ii) that the abundance of miRNAs is correlated with platelet activation.

## Materials and Methods

### Study Design

In the current study, plasma samples obtained in a previously conducted single-center, parallel-group randomized controlled trial (Laky et al., 2018) were used to test whether the applied treatment affected the abundance of several miRNAs. A detailed description of the trial design, including a CONSORT flow diagram (Moher et al., 2010), can be found in the respective publication (Laky et al., 2018). In short, patients with newly diagnosed periodontitis were randomized to either receive intensive periodontal treatment (treatment group) or community-based periodontal care (control group). The study design was not changed throughout the study and was approved by the institutional review board of the Medical University of Vienna, Austria (vote 1656/2014).

### Participants and Setting

Patients were included after informed consent if they understood of the study requirements, had at least one interproximal site with a probing depth ≥ 5 mm and a loss of attachment at ≥ 2 interproximal sites ≥ 5 mm. Exclusion criteria comprised all systemic diseases including diabetes mellitus, chronic renal failure or liver cirrhosis, systemic antibiotic treatment in the preceding 3 months, periodontal treatment within the last 4 months, pregnancy or breastfeeding, and any medication affecting platelets. Examinations, treatments, and blood samplings took place at the Division of Conservative Dentistry and Periodontology, School of Dentistry, Medical University of Vienna.

### Interventions

Patients randomized to the intervention group received intensive periodontal treatment, consisting of subgingival debridement with curettes and sonic instruments, as described previously in detail (Laky et al., 2018). Patients of the control group received community-based periodontal care.

### Outcomes

#### Primary and Secondary Outcome Measures

All primary and secondary outcomes were published previously and showed that intensive periodontal treatment reduces platelet activation compared to community-based periodontal care (Laky et al., 2018).

The primary outcome measure according to the study protocol was platelet P-selectin expression in the absence of a platelet agonist, reflecting the basal activation state of platelets *in vivo*. Secondary outcome measures included basal platelet activation as measured by additional platelet activation markers such as CD40L expression and GPIIb/IIIa activation. In addition, platelet function was tested by adding the platelet agonist ADP in different concentrations to quantify the tendency of platelets to become activated by a given stimulus, i.e., platelet reactivity. Both P-selectin expression and the other platelet activation markers used to assess basal platelet activation served as readouts for platelet reactivity.

### Outcome Measures Used in the Current Study

The abundance of miRNAs in plasma served as outcome measures in the current study. We selected a panel of miRNAs which might be used to predict platelet function according to previous research. In addition, we included miRNAs as negative control which should be independent of platelet function. Table 1 in one of our previous studies (Mussbacher et al., 2020) on miRNA analyses gives an overview of the miRNAs chosen for this study, together with a short summary of the respective previous research.

### Sample Size, Randomization and Blinding

Sample size and randomization of the present study was defined by the previously published study (Laky et al., 2018). Two patients had to be excluded in the current analysis due to hemolysis. All persons analyzing the abundance of miRNAs in plasma samples were blinded from the respective patient’s identity, and thus group allocation.

### Quantification of miRNAs

#### Blood Sampling and Preparation of Platelet-Poor Plasma

Venous blood was drawn using a 20 G cannula and was anti-coagulated with CTAD (citrate, theophylline, adenosine, dipyridamole) or citrate. Immediately after blood sampling, tubes were put on ice to minimize *ex vivo* platelet activation. Thereafter, anti-coagulated blood was centrifuged for 10 min with 1000 × *g* at 4°C. Only the upper two thirds of supernatant were removed to minimize leukocyte contamination and centrifuged again for 10 min with 10 000 × *g* at 4°C to remove all cellular components.

### RNA Extraction and Quantitative Real-Time Polymerase Chain Reaction (qPCR)

The miRNeasy Mini Kit (QIAGEN, Germany) was applied to isolate total RNA, including small RNAs, from plasma. qPCR analysis was performed as described previously (Mussbacher et al., 2020). Robustness of RNA extraction, complementary DNA synthesis, and qPCR amplification was assessed using combinations of synthetic spike-in controls (Exiqon, Denmark). Hemolysis was assessed using the ratios of miRNA-23a-3p and miRNA-451a and positive samples were excluded from the analysis.

### Platelet Function Tests

Platelet function tests were previously described in detail (Laky et al., 2018).

### *In vitro* Study

Within 30 min after blood drawing, citrated blood was centrifuged for 20 min with 125 × *g* at room temperature to generate platelet-rich plasma (PRP). Platelets were stimulated in PRP with different concentrations of adenosine diphosphate (ADP) for 10 min at room temperature to trigger platelet activation *in vitro*.

### Statistical Methods

Generally, statistical analyses were performed using IBM SPSS statistics 26. The primary outcome parameter of this study was published already. There was no correction for multiple testing in addition to the ones mentioned below. Results have therefore to be interpreted accordingly. Graphs were generated using GraphPad Prism 8.3. *P*-values ≤ 0.05 were considered statistically significant.

#### Effects of Intensive Periodontal Treatment on miRNA Abundance

To estimate the treatment effects on miRNAs, separate analyses of covariance (ANCOVA) were applied for each miRNA. Each model included the binary between-subjects factor “group” with the levels “intervention” and “control.” Further, the baseline miRNA-levels of each patient were included as covariate. The Cq-values of each miRNA, normalized to an RNA spike-in control, were used as dependent variables. In this way, the group difference after 3 months could be estimated while adjusting for baseline differences. Approximate normal distribution of errors were checked visually. Homogeneity of regression slopes was inspected visually and tested by including a “group × baseline values” interaction term. Only interaction terms were considered significant that showed a *p* value < 0.05 after correction for 16 interaction terms according to Bonferroni-Holm. In addition to each individual’s time course of Cq-value change, the estimated group difference after 3 months, representing the treatment effect, was plotted with 95% confidence intervals and the corresponding *p*-value. To assess whether effects might have been affected by platelet count and platelet RNA content, the respective values were entered as additional covariates in separately reported statistical models.

#### Relationship Between miRNA Abundance and Basal Platelet P-selectin Expression

To assess how closely the abundance of quantified miRNAs in plasma samples are related to basal P-selectin expression sampled at the same time point, we used the following regression model. P-selectin expression was used as continuous predictor (covariate), the experimental group as the factor “group” with the levels “intervention” and “control,” and the respective miRNA abundance as dependent variable. At first, a “group × P-selectin” interaction was tested, as the slope of regression lines describing the relationship between basal P-selectin expression and miRNA abundance might be different between groups after periodontal treatment. Interactions with *p*-values > 0.05 were omitted from the model, whereby the interaction p-values were corrected for multiple testing of 16 interactions according to Bonferroni-Holm. Next, a main effect of the factor “group” was tested, which would indicate that the regression lines would be parallel shifted. In analogy with the interaction term, main effects with *p*-values > 0.05 were omitted from the model, leaving only P-selectin as the only predictor for miRNA abundance in the model. The square root of the *R*^2^-value, corresponding to the Pearson r correlation coefficient, was taken as the measure for correlation.

For each miRNA, this regression model was used separately for data acquired after 3 months, and for the change between baseline and 3 months. For the latter, 3 months - baseline P-selectin expression was used as predictor, and 3 months - baseline miRNA abundance as dependent variables. A third regression model was applied for the baseline values, where the factor group was not included, as before randomization there were no groups. Scatter plots are depicted for each miRNA at each time point with the respective regression line(s) with their 95% confidence intervals and corresponding p-values.

In additional models, reported separately, platelet count and platelet RNA content were entered as additional predictors to assess their influence on the results.

#### Correlation Between Alternative Platelet Activation Markers and Platelet Reactivity

To explore whether other platelet activation markers might correlate in a monotone manner with certain miRNAs, Spearman correlation coefficients were used, implicitly assuming that the correlations were not different between groups at all time points. To avoid a multitude of scatter plots, the correlation coefficient with its 95% confidence interval is shown.

#### *In vitro* Analyses of Platelet Activation With miRNA Release

To test whether activating platelets with ADP results in platelet activation and at the same time to miRNA release, mixed linear models were used. Different ADP concentrations were used as fixed effect in its continuous form, each volunteer as level of a random factor to account for the correlation within subjects. Platelet P-selectin expression or any of the measured miRNAs were used as dependent variables.

#### Statistical Adjustment for Platelet Count and RNA Content Did Not Change Results

As one might argue that platelet count and platelet RNA content might have affected the results, we performed ancillary analyses that additionally adjust for differences in platelet count as well as platelet RNA content after 3 months. However, adjustment did not alter the results to a relevant extent (Supplementary Table 1).

## Results

### Periodontal Treatment Modulates Platelet Function, Yet Not the Abundance of miRNAs in Plasma

**FIGURE 1:**
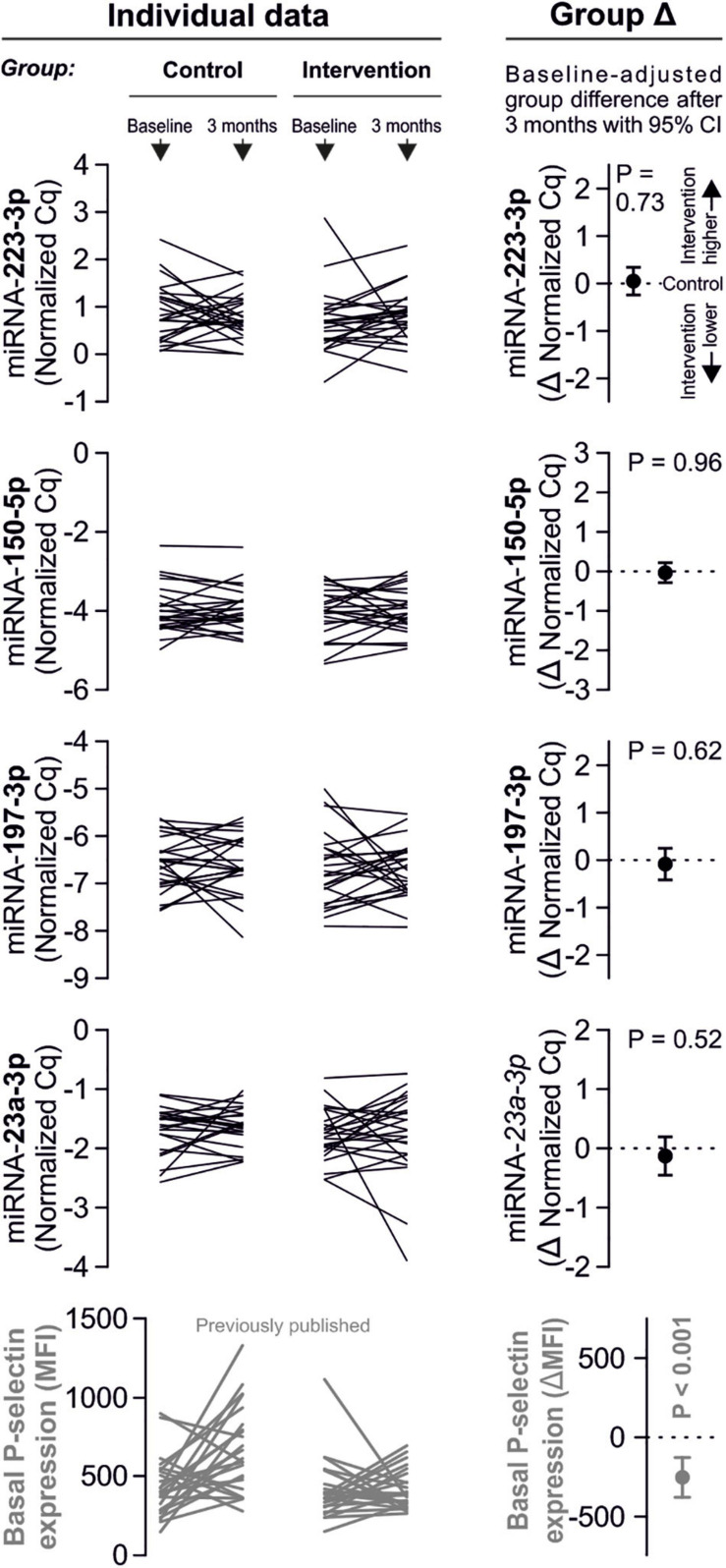
No effects of periodontal treatment on platelet-related miRNAs. A panel of miRNAs was quantified in plasma samples from patients with periodontal disease enrolled in a randomized controlled trial to test the effects of periodontal treatment on platelet function. Although the intervention reduced platelet activation measured by platelet surface P-selectin expression [published previously in Laky et al. (2018)], no relevant change of platelet microRNAs could be detected.

**FIGURE 2:**
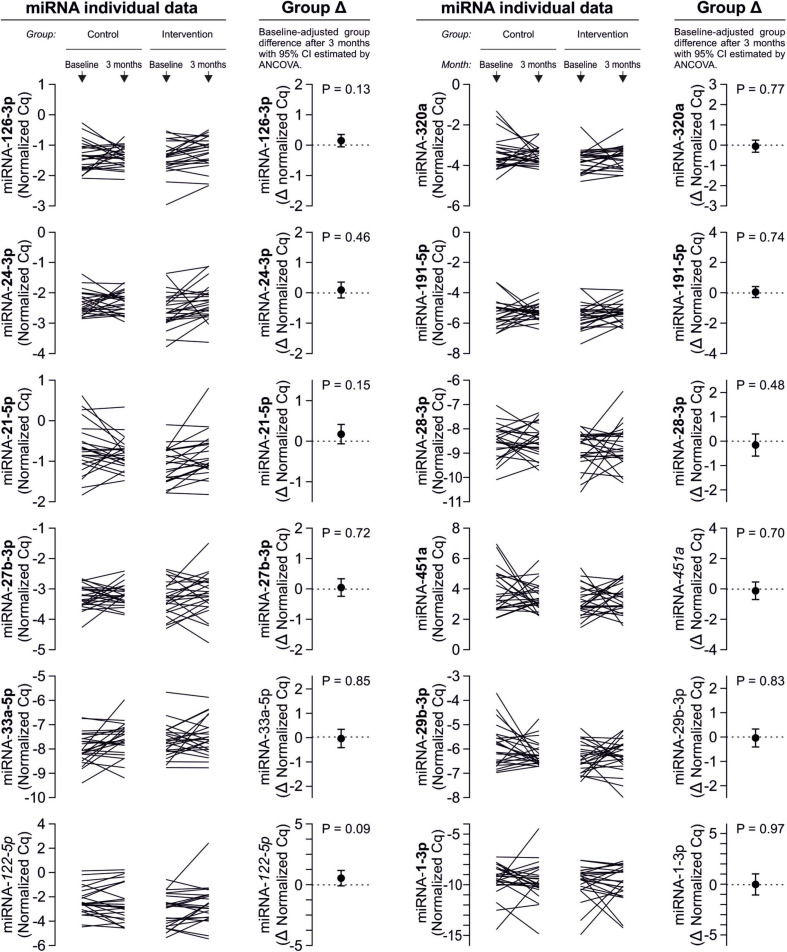
No effects of periodontal treatment on additional platelet-related miRNAs. Additionally quantified miRNAs showed no relevant change caused by the intervention that affected platelet activation. miRNA 122-5p serves as platelet-independent control.

Despite clear effects of periodontal treatment on platelet activation, we found no relevant effects on several plasma miRNAs in this study. Neither miRNA 223-3p, 150-5p, 197-3p nor 23a-23p were affected to a relevant extent (Figure 1). Similarly, several other miRNA species remained more or less unchanged (Figure 2). Based on literature (Zampetaki et al., 2012), the treatment effects on miRNA 126-3p were also adjusted for miRNA 223-3p and 197-3p, which resulted in no relevant change of the estimate (Supplementary Figure 1).

### No Correlation Between miRNA Abundance and Platelet Activation Quantified by P-selectin Expression

**FIGURE 3:**
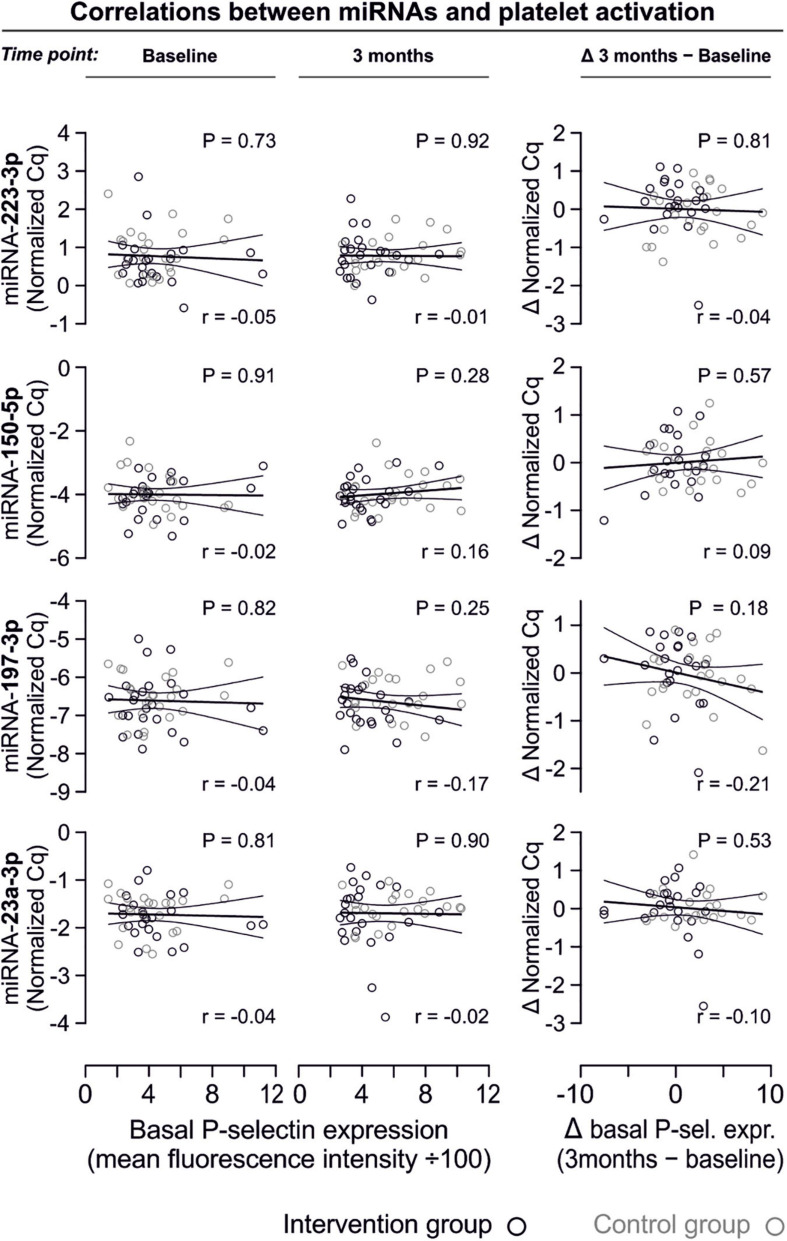
No relevant correlation between platelet-microRNAs and platelet activation. Platelet activation was determined by quantifying platelet surface P-selectin expression with fluorophore-conjugated antibodies, fluorescence is plotted on the horizontal axes. The *P*-values refer to the hypotheses that the slopes of the plotted regression lines are different from 0, i.e., non-horizontal and that there is a non-zero correlation between P-selectin expression and miRNA abundance. The error bands are 95% confidence intervals for the regression lines. No correlations between miRNAs and platelet activation were observed, neither at the initial examination nor after the intervention period. The change of platelet activation throughout the observational period (Δ basal P-selectin expression) was also not correlated with changes of miRNAs.

**FIGURE 4:**
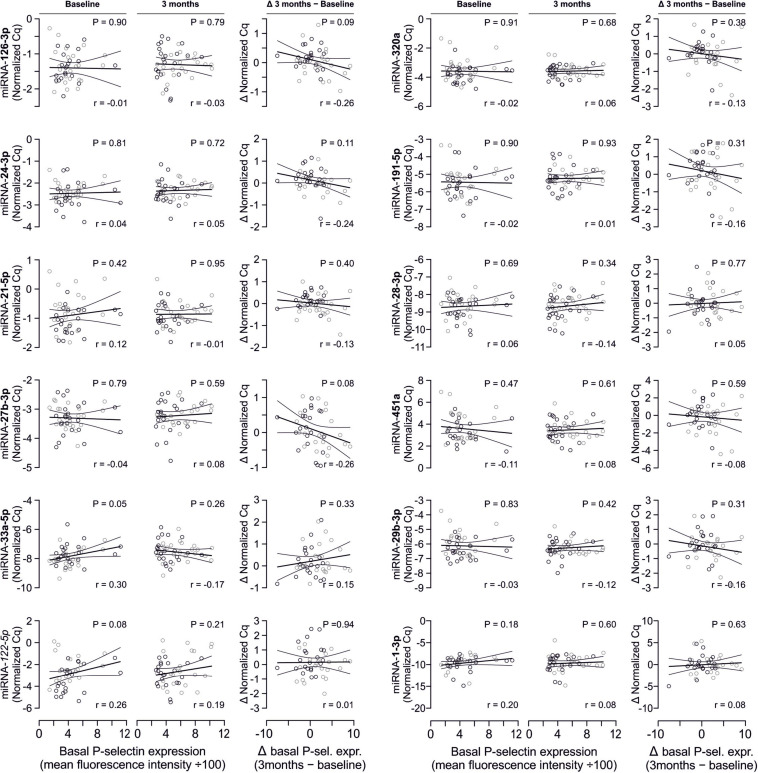
No relevant correlation between additional platelet-related microRNAs and platelet activation. Additionally quantified miRNAs showed no relevant association with platelet activation at any time point or with the change of platelet activation.

In line with the above-stated results there were no relevant correlations between miRNA abundance and basal platelet activation measured by surface P-selectin expression. To ensure that no relevant correlations have been overlooked, correlation coefficients were not only estimated for each of the two time points (baseline, 3 months), but also between the intra-individual changes in miRNA abundance and changes in platelet activation. Neither of these strategies revealed any relevant correlations (Figures 3, 4). The relationship between miRNA 126-3p and platelet function was also not altered after adjustment for miRNA 223-3p and 197-3p (Supplementary Table 1).

### No Correlation Between miRNA Abundance and Platelet Activation and Reactivity Quantified by a Panel of Activation Markers

**FIGURE 5:**
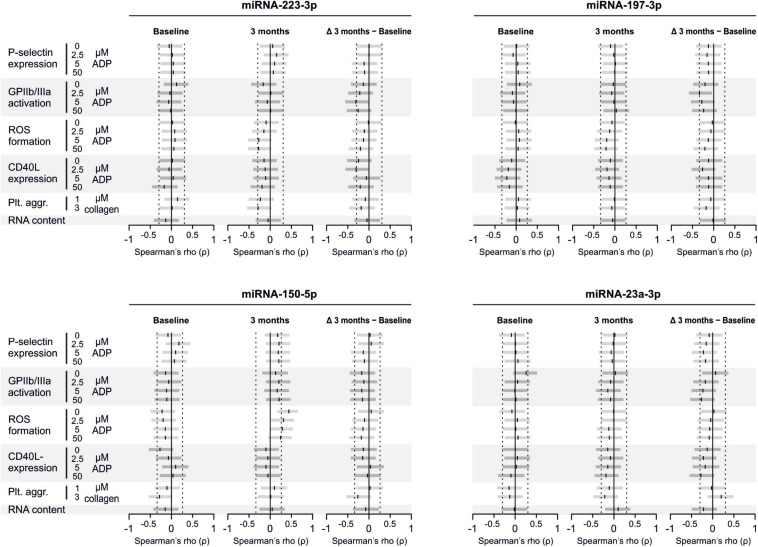
Relationship between platelet activation/reactivity as measured by several established markers and plasma miRNA abundance. Monotone correlations quantified by Spearman rank correlation coefficients between basal platelet activation (0 μM ADP) and platelet reactivity (2.5, 5, and 50 μM ADP) as measured by distinct platelet activation markers. Each small vertical black stroke indicates the point estimate of the respective correlation coefficient. A point estimate of 1 would indicate a perfect monotone positive correlation and –1 a negative one. The vertical dotted lines at rho = –0.3 and +0.3 delimit correlations considered to be irrelevant, 0 indicates absence of correlation. The horizontal gray bars shows the 95% confidence intervals of the correlation coefficients without correction for multiple testing. They can be interpreted insofar as our data are compatible with all values within the error bar. Note that most error bars are largely located within rho ranges considered irrelevant.

**FIGURE 6:**
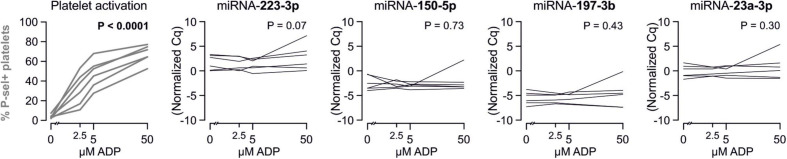
Relationship between platelet activation/reactivity as measured by several established markers and plasma abundance of additional miRNAs. Detailed description of Figure 5 applies.

**FIGURE 7:**
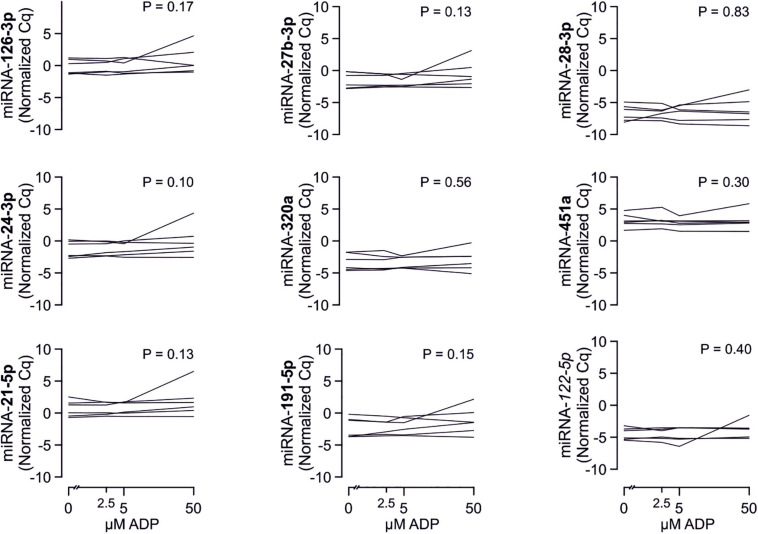
Relationship between platelet activation/reactivity as measured by several established markers and plasma abundance of additional miRNAs. Detailed description of Figure 5 applies.

There are several aspects of platelet activation, which are related to each other, but nevertheless represent distinct functions. Thus, we explored whether miRNAs quantified in patient plasma might correlate with platelet activation quantified by several other markers. In addition, we assessed whether miRNAs might correlate with platelet reactivity, measured quantification of activation markers after *in vitro* platelet activation. However, miRNAs were unable to predict any aspect of platelet activation or reactivity (Figures 5–7).

### Analyzed miRNAs Are Not Released by Platelets Upon Activation

**FIGURE 8:**
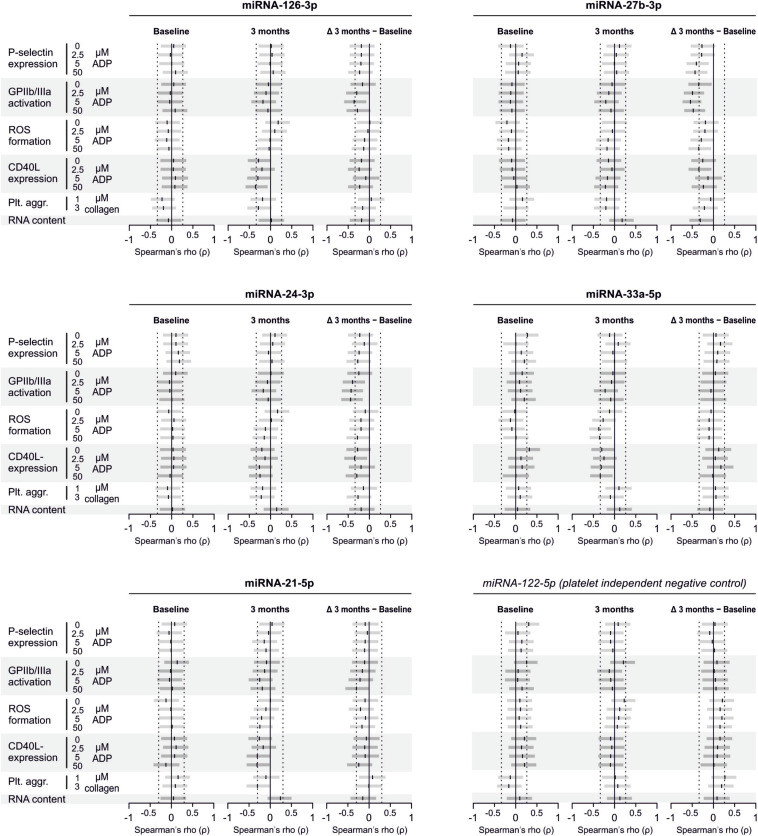
No detection of miRNA release by platelets from healthy volunteers. Platelets from six healthy volunteers were activated by ADP (left, gray). miRNAs were determined in the supernatant, which can be assumed to contain molecules released by platelets.

**FIGURE 9:**
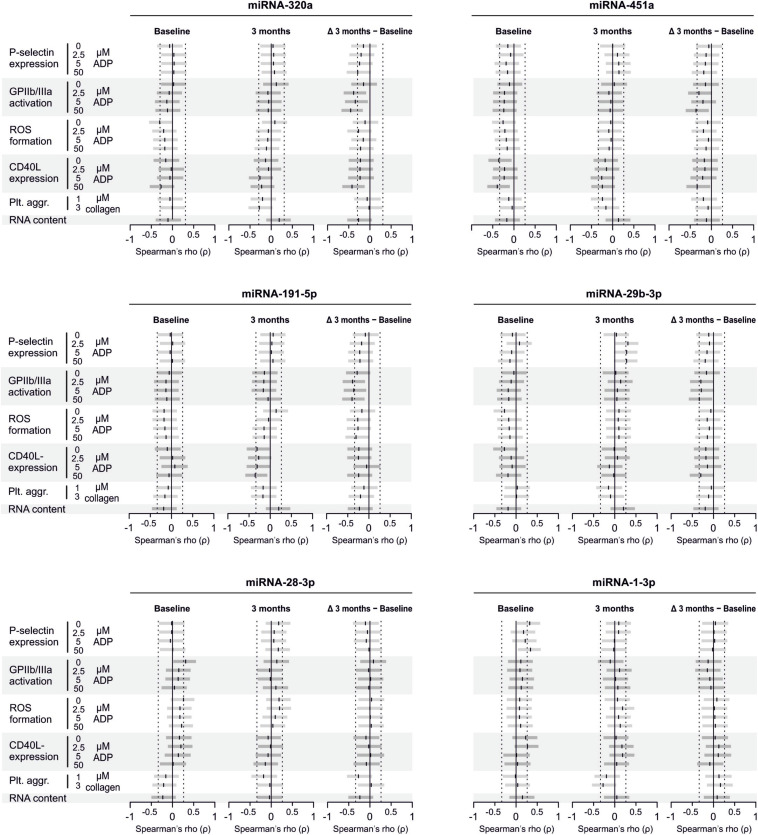
No release of additional miRNAs by platelets from healthy volunteers. Platelets from six healthy volunteers were activated by ADP (left, gray). miRNAs were determined in the supernatant, which can be assumed to contain molecules released by platelets.

**FIGURE 10:**
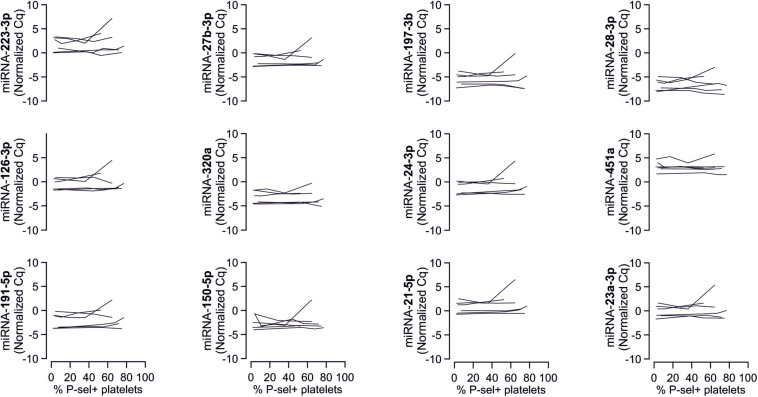
No association between P-selectin expression of ADP-activated platelets sampled from healthy volunteers and the amount of mRNAs in the respective supernatants. Plots show the relationship between ADP-induced P-selection expression and miRNA abundance in the supernatant.

To confirm that platelets release some of the investigated miRNAs - a property that would make them especially promising as potential biomarker - we sampled whole blood from six healthy volunteers and quantified miRNAs in the supernatant of resting platelets or ones that were activated by different concentrations of ADP *in vitro*. Based on the percentage of platelets expressing P-selectin, addition of different concentrations of ADP activated platelets as expected (*p* < 0.0001). In contrast, there was no relevant increase of platelet miRNA abundance in the supernatant of activated platelets compared to the supernatant of non-activated platelets, suggesting that platelets do not release relevant amounts of these miRNAs upon activation (Figures 8–10).

## Discussion

This study was performed to corroborate previous findings that certain miRNAs might be used as biomarkers for *in vivo* platelet activation. For this purpose, we analyzed plasma samples obtained in a previously conducted randomized controlled trial in which periodontal treatment was effective in modulating platelet function (Laky et al., 2018). However, despite the clear effects of periodontal treatment on platelet activation, we found no relevant effects on several plasma miRNAs in this study. Moreover, miRNAs showed no relevant correlation with platelet activation. Further, our results indicate that the investigated miRNAs are not released by platelets upon activation.

An underlying concept of platelet miRNAs as biomarkers for platelet function is that platelets store miRNAs and release them upon activation. Based on that, the abundance of these miRNAs is expected to correlate with *in vivo* platelet activation and might thus serve as biomarker. Among others, this was shown for miRNA-126 and miRNA-223. Platelet-poor plasma was spiked with various amounts of isolated platelets, whereupon miRNA abundance was quantified in the resulting mixture. Results showed a dose-dependent increase on miRNA-126 and miRNA-223 abundance, suggesting that these miRNAs are in fact stored by platelets (Kaudewitz et al., 2016). Further evidence comes from a small dose-escalating study in healthy volunteers, where miRNA abundance was quantified at baseline, after one week of prasugrel 10 mg, after another week of continued prasugrel 10 mg with additional ASA 75 mg, and after a last week with prasugrel 10 mg combined with ASA 300 mg. With each week the abundance of miRNA126 and miRNA-223 continued to decrease. In parallel, patients suffering from symptomatic carotid atherosclerotic disease on ASA 75 mg treatment were given additional high doses of dipyridamole or clopidogrel, which was followed by an abundance decrease of these two miRNAs (Willeit et al., 2013). Another study reported positive correlations both for miRNA-126 and miRNA-223 with two distinct platelet function tests (Sunderland et al., 2017). Furthermore, a study (Zampetaki et al., 2012) showed that miRNA 126 is only predictive of myocardial infarctions after adjustment for miRNA 223 and 197. Therefore, we explored whether the treatment-effects or the correlations between miRNA 126 and platelet function might be altered after adjustment for the miRNA 223 and 197. However, these analyses provided no indication that adjustment had any relevant effect.

The above-mentioned concept can be rejected with sufficient probability for the investigated miRNAs. Notably, this does not necessarily apply to other miRNAs that were not investigated in this study. However, we focused our investigation on miRNAs for which literature provided sufficient evidence. An overview over the physiological roles including the reason for our miRNA selection can be found in our previous publication on the optimal sampling preparation of platelets for miRNA analyses (Mussbacher et al., 2020).

As there are distinct aspects of platelet activation, as, to name a few, conformational change of the fibrin receptor GPIIb/IIIa causing a high-affinity state as a prerequisite for activation, ROS production, or degranulation, we correlated the abundance of miRNAs with these parameters and also found no correlation. Therefore, it seems unlikely that we have overlooked a certain aspect of platelet activation that could be predicted by circulating miRNAs.

Although our evidence clearly supports the view that these miRNAs are unsuitable as markers for *in vivo* platelet activation, one has to acknowledge that there are some limiting factors that apply to our study. First, the sample size is rather small compared to some other correlational studies (Kaudewitz et al., 2016). It is possible that the predictive applicability of miRNAs is only given when they are used simultaneously in a multivariable regression model. This needs to be addressed in future studies with a sample size adequate for this purpose. However, the sample size was adequate to detect the difference between groups in the primary outcome. Furthermore, some degree of uncertainty remains regarding association strengths. Strictly speaking, many 95% confidence intervals of correlation coefficients cover a range from irrelevant to interesting. Specifically, those correlation coefficients higher/lower than 0.3/−0.3 with a confidence interval covering irrelevant to relevant correlation strengths remain as possible correlations and warrant further research. Thus, based on our data we cannot claim that correlation coefficients are in the range of zero. Also, the multiplicity of analyses has to be stressed. As *p*-values and corresponding confidence intervals were not adjusted, approximately 5% of all correlation coefficients must be expected to be associated with a “significant” *p*-value below 0.05, i.e., to be false positive under the null hypothesis. Therefore, it is important that our data are not interpreted in terms of strong evidence against relevant correlations. They merely do not constitute further evidence for such correlations. Another limitation refers to generalizability of our findings. It is conceivable that specifically in our cohort suffering from periodontal disease, miRNAs are less suitable as biomarkers for platelet activation than in other cohorts. In this regard, it needs to be mentioned that our study did not include a control group of healthy subjects. One could speculate that miRNA abundance indeed differs between healthy controls and those with periodontal disease.

In conclusion, we could not corroborate the applicability of certain miRNAs as biomarkers for platelet function in a specific cohort suffering from periodontal disease. It is important to emphasize that our data do not generally rule out miRNAs as biomarkers for platelet function. However, we consider our results a small yet important piece of evidence that should be given attention in future discussions concerning miRNAs as biomarkers.

## Data Availability Statement

The raw data supporting the conclusion of this article will be made available by the authors, without undue reservation.

## Ethics Statement

The studies involving human participants were reviewed and approved by Institutional review board of the Medical University of Vienna, Austria (Ethikkommission der Medizinischen Universität Wien). The patients/participants provided their written informed consent to participate in this study.

## Author Contributions

SH, IA, LW, TK, HH, WS, MH, and AA performed the experiments. SH, ML, WS, MH, AM, IV, and AA analyzed the results. SH and AA prepared the figures, designed the research, and wrote the manuscript. All authors contributed to the article and approved the submitted version.

## Conflict of Interest

TK was employed by TAmiRNA GmbH. MH was employed by and owns stock in TAmiRNA GmbH. The remaining authors declare that the research was conducted in the absence of any commercial or financial relationships that could be construed as a potential conflict of interest.
