# Supplementary material for: Platelet Activation Is Not Always Associated With Platelet-Related Plasma microRNA Abundance – Results From a Randomized Controlled Trial of Periodontal Patients
Source: Front Physiol. 2021 Mar 1;12:613515. doi: 10.3389/fphys.2021.613515 (PMC7957016; doi:10.3389/fphys.2021.613515)
Supplement: Supplementary file 1 [file Data_Sheet_1.PDF]

frontiers

ONLINE SUPPLEMENT

Platelet activation is not always associated with platelet-related plasma microRNA abundance – results from a randomized controlled trial of periodontal patients

Stefan Heber[1], Markus Laky[2], Isabella Anscheringer[3], Lukas Wolschner[2], Marion Mussbacher[3], Teresa Krammer[4], Hady Haririan[2], Waltraud C. Schrottmaier[3], Ivo Volf[1], Matthias Hackl[4], Andreas Moritz[2], Alice Assinger[3]

The following additional analyses were carried out to test whether adjustment for miR-223 and -197 might affect the relationship between platelet function and miRNAs. These analyses were performed as data of Zampetaki et al. [1] suggest that miRNA-126 is only predictive of myocardial infarctions after adjustment for miRNA-223 and -197.

1 Re-estimation of the periodontal treatment effect on miRNAs with adjustment

miR-223 and -197 were entered as additional covariates in the model testing the treatment effect on miRNAs. For each of the two adjustment miRNAs, two covariates were used, one to hold the baseline value constant and one to hold the post-treatment value constant. Results (Suppl. Fig. 1) shows that adjustment did not alter the results to a relevant extent.

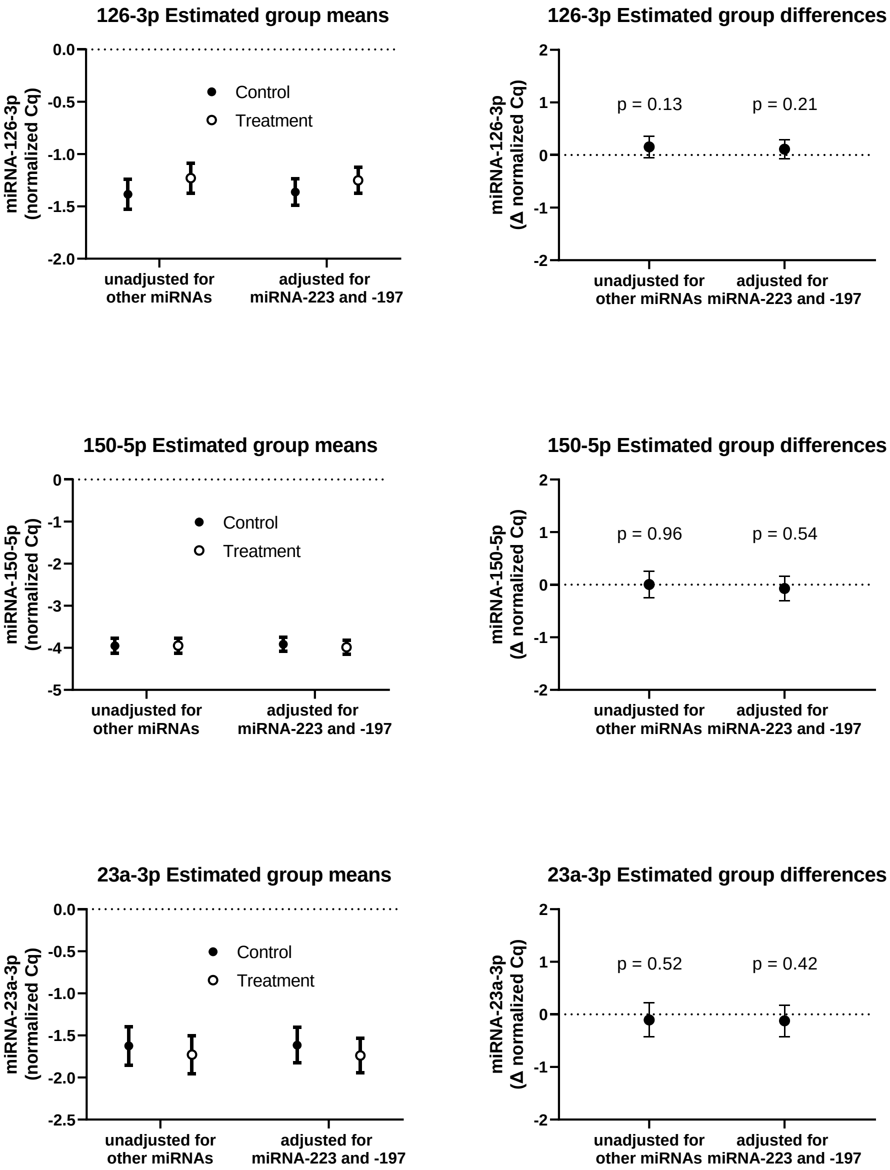

Suppl. Fig. 1. Effect of adjustment for miRNA-223 and -197 on estimated miRNA-126, -150-5p and -23a-3p levels. The unadjusted results are already shown in the main manuscript and are intended to facilitate comparison.

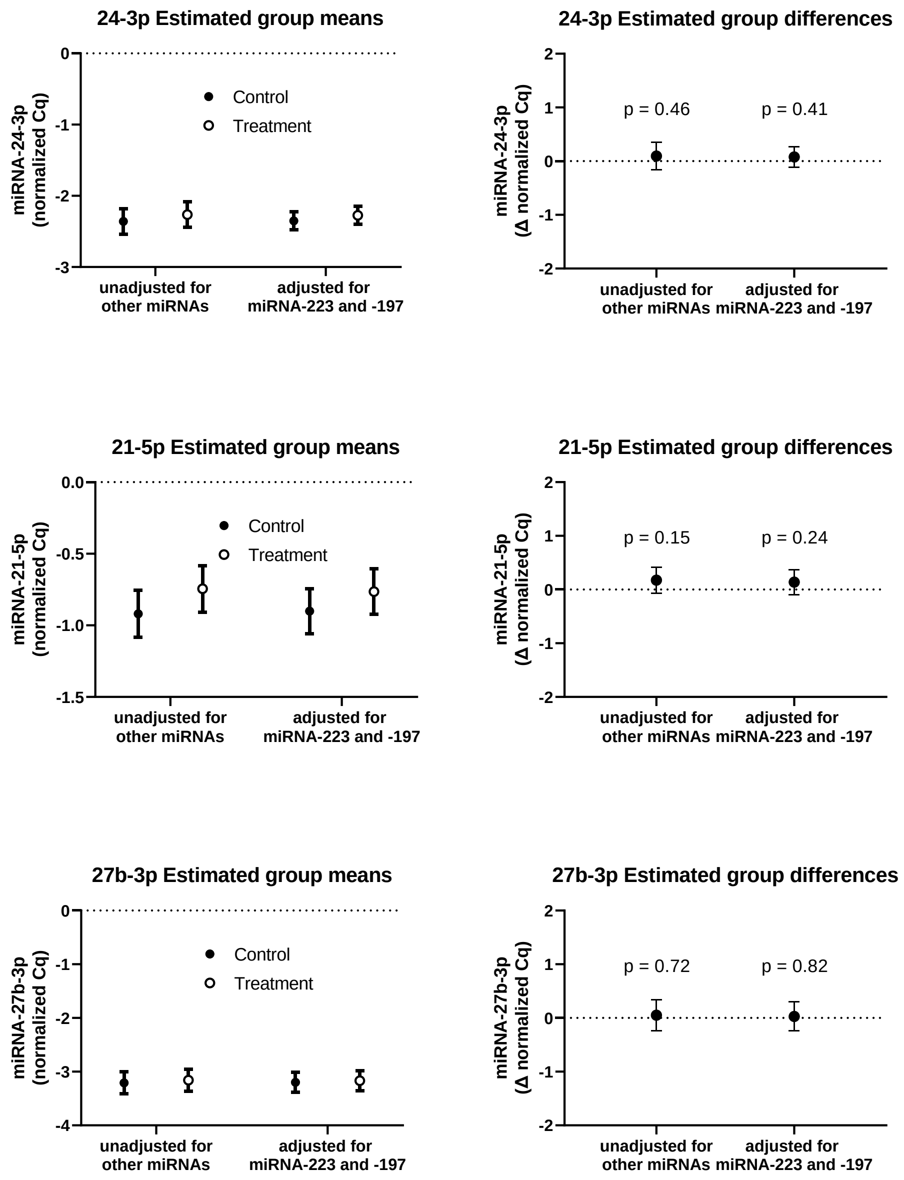

Suppl. Fig. 2. Effect of adjustment for miRNA-223 and -197 on estimated miRNA-24-3p, -21-5p and -27b-3p levels. The unadjusted results are already shown in the main manuscript and are intended to facilitate comparison.

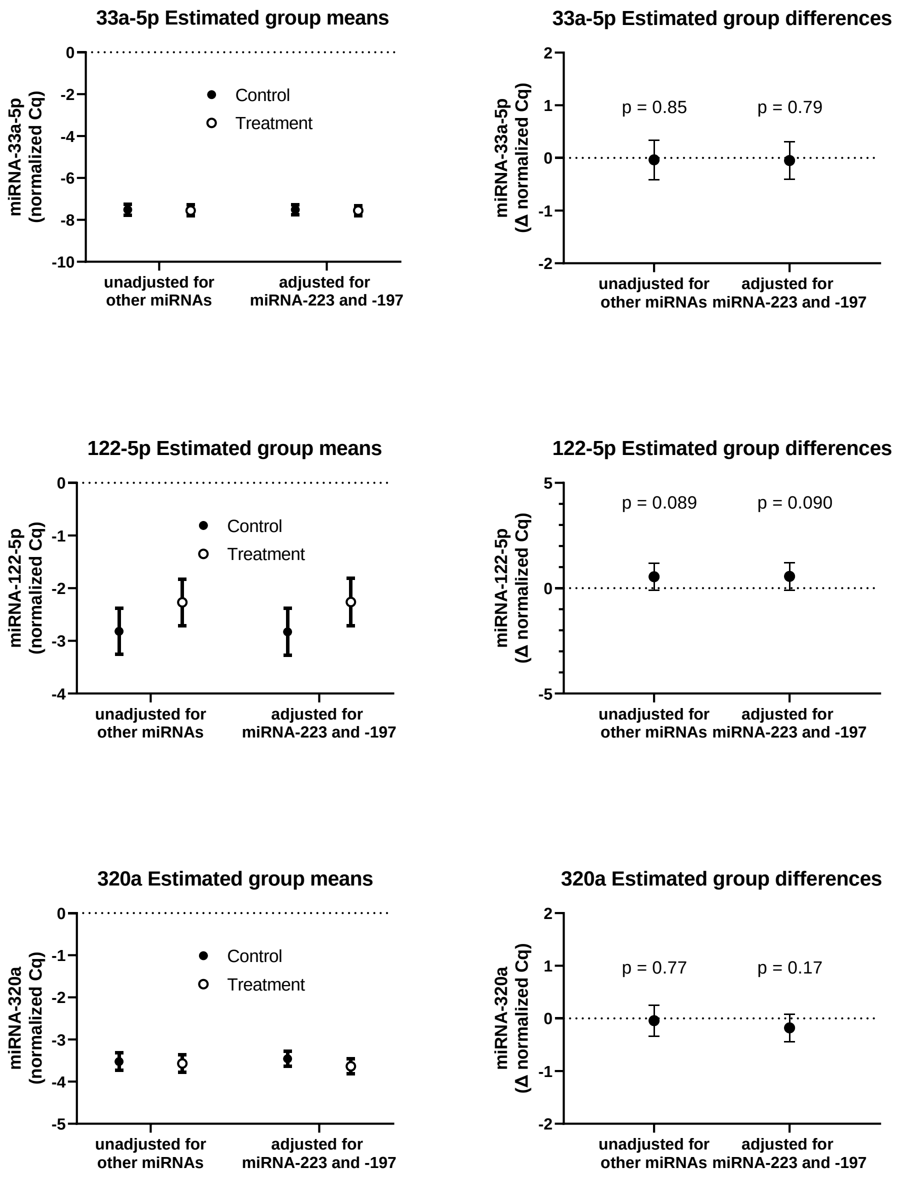

Suppl. Fig. 3. Effect of adjustment for miRNA-223 and -197 on estimated miRNA-33a-5p, -122-5p and -320a levels. The unadjusted results are already shown in the main manuscript and are intended to facilitate comparison.

This is a provisional file, not the final typeset article

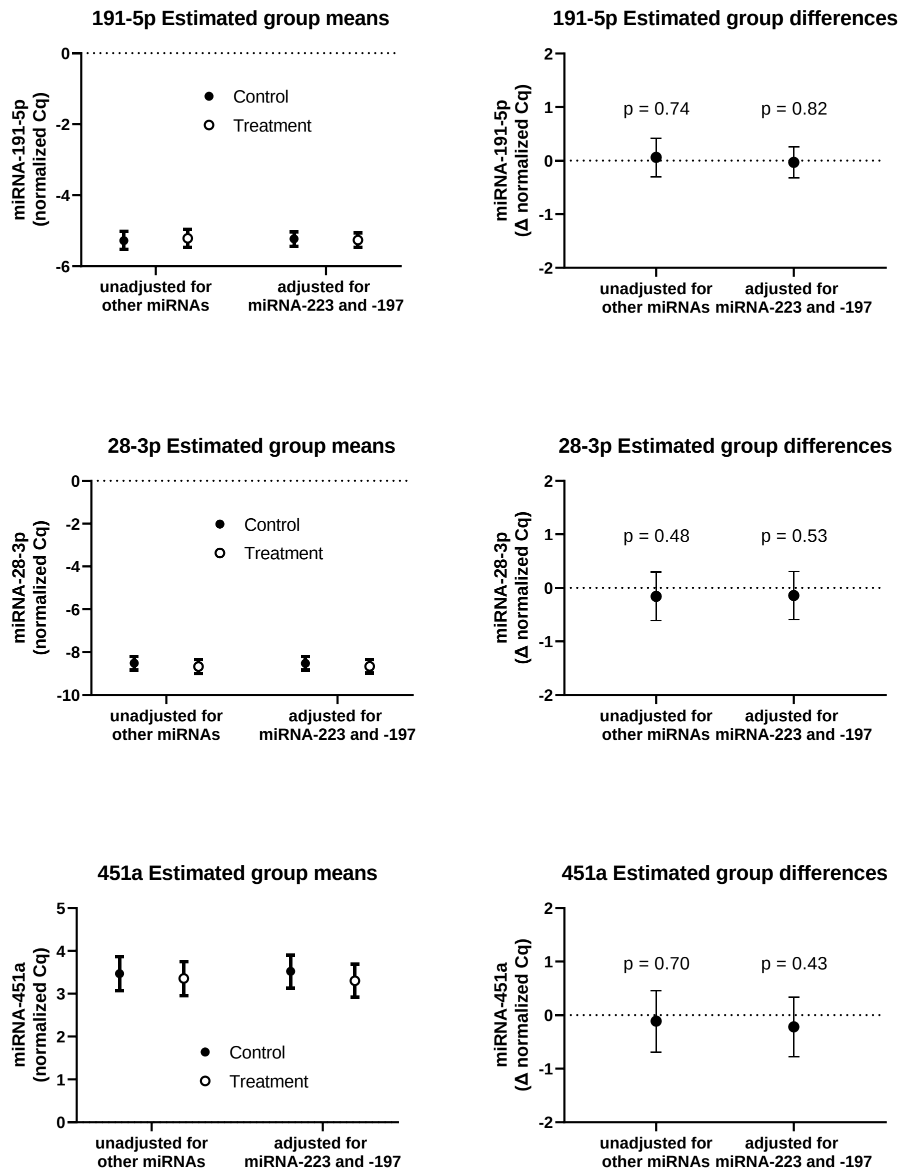

Suppl. Fig. 4. Effect of adjustment for miRNA-223 and -197 on estimated miRNA-191-5p, -28-3p and -451a levels. The unadjusted results are already shown in the main manuscript and are intended to facilitate comparison.

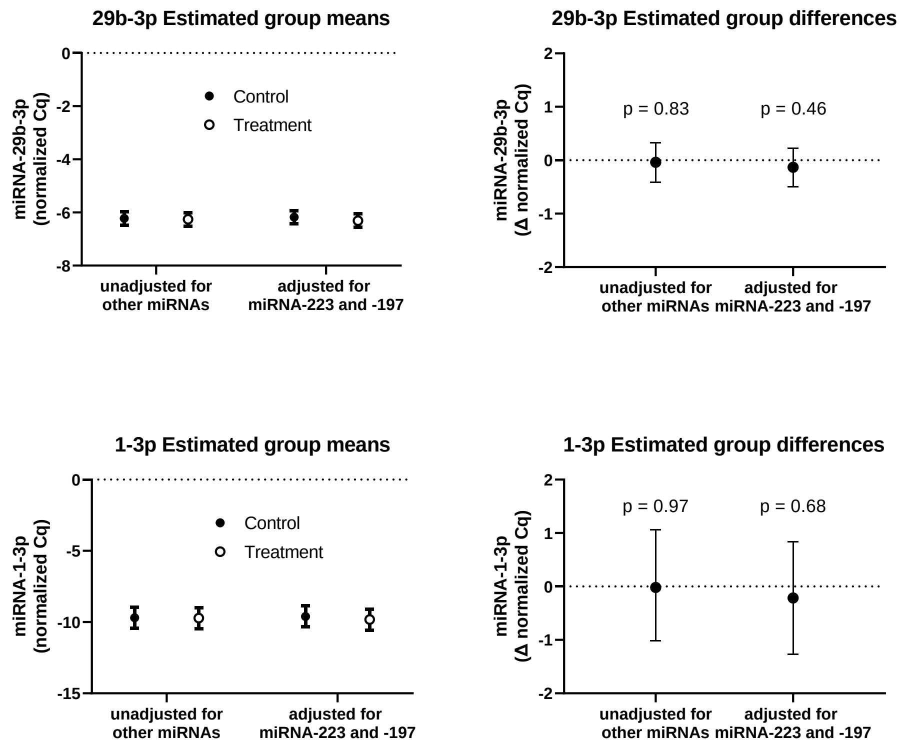

Suppl. Fig. 5. Effect of adjustment for miRNA-223 and -197 on estimated miRNA-29b-3p and 1-3p levels. The unadjusted results are already shown in the main manuscript and are intended to facilitate comparison.

2 Re-estimation of correlations between miRNAs and platelet function with adjustment

Analogous to analyses shown in Fig. 2, the correlation coefficients (r) with their corresponding p-values were estimated using data obtained at baseline, after 3 months and their difference. The predictors were basal P-selectin expression, miRNA-223 and-197. MiRNAs listed in the left column were used as dependent variable. As shown in Suppl. Table 1, adjustment did not alter the relationships at each time point to a relevant extent.

However, there was a weak, yet significant negative correlation between the change of platelet function and the change of miRNA-126 after adjustment the change of miRNA-223 and -197. The additional and exploratory nature of the analysis should be kept in mind. Nevertheless, considering the role of activated platelets in the onset of myocardial infarctions, only a positive relationship would be in line with Zampetaki's results. A correlation of similar magnitude was observed for miRNA 24-3p, however, also indicating a negative relationship. The only other miRNA that exceeded the threshold of statistical significance was miRNA 33a-5p. Thereby it has to be noted that the correlation is still rather low and of no value for a biomarker. In addition, if 33a-5p was a reliable marker reflecting platelet activation, a similar correlation should have occurred after 3 months.

This is a provisional file, not the final typeset article

Suppl. Table 1. Effect of adjustment for miRNA-223 and -197 on correlations between basal platelet activation (P-selectin) and miRNA levels.

		**BASELINE**		**3 MONTHS**		**Δ 3 MO - BL**	
miRNA		r	p	r	p	r	p
126-3p	Unadjusted	-0.01	0.90	-0.03	0.79	-0.26	0.09
	Adjusted	0.02	0.94	-0.09	0.56	**-0.31**	**0.043**
150-5p	Unadjusted	-0.02	0.91	0.16	0.28	0.09	0.57
	Adjusted	0.00	0.98	0.13	0.39	0.11	0.49
23a-3p	Unadjusted	-0.04	0.81	-0.02	0.90	-0.10	0.53
	Adjusted	-0.01	0.98	-0.02	0.88	-0.07	0.69
24-3p	Unadjusted	0.04	0.81	0.05	0.72	-0.24	0.11
	Adjusted	0.14	0.36	0.09	0.56	**-0.32**	**0.040**
21-5p	Unadjusted	0.12	0.42	-0.01	0.95	-0.13	0.40
	Adjusted	0.18	0.23	0.03	0.86	-0.14	0.38
27b-3p	Unadjusted	-0.04	0.79	0.08	0.59	-0.26	0.08
	Adjusted	-0.01	0.94	0.09	0.56	-0.26	0.09
33a-5p	Unadjusted	0.30	0.05	-0.17	0.26	0.15	0.33
	Adjusted	**0.36**	**0.016**	-0.22	0.14	0.17	0.28
122-5p	Unadjusted	0.26	0.08	0.19	0.21	0.01	0.94
	Adjusted	0.29	0.06	0.21	0.16	0.03	0.85
320a	Unadjusted	-0.02	0.91	0.06	0.68	-0.13	0.38

	Adjusted	0.02	0.90	0.04	0.79	-0.17	0.28
191-5p	Unadjusted	-0.02	0.90	0.01	0.93	-0.16	0.31
	Adjusted	0.02	0.90	-0.02	0.91	-0.21	0.19
28b-3p	Unadjusted	0.06	0.69	-0.14	0.34	0.05	0.77
	Adjusted	0.08	0.62	0.18	0.25	-0.01	0.94
451a	Unadjusted	-0.11	0.47	0.08	0.61	-0.08	0.59
	Adjusted	-0.10	0.54	0.04	0.78	-0.11	0.49
29b-3p	Unadjusted	-0.03	0.83	-0.12	0.42	-0.16	0.31
	Adjusted	-0.01	0.96	0.06	0.69	-0.19	0.22
1-3p	Unadjusted	0.20	0.18	0.08	0.60	0.08	0.63
	Adjusted	0.22	0.15	0.00	0.99	0.10	0.51

[1] A. Zampetaki, P. Willeit, L. Tilling, I. Drozdov, M. Prokopi, J.M. Renard, A. Mayr, S. Weger, G. Schett, A. Shah, C.M. Boulanger, J. Willeit, P.J. Chowienczyk, S. Kiechl, and M. Mayr, Prospective study on circulating MicroRNAs and risk of myocardial infarction. Journal of the American College of Cardiology 60 (2012) 290-9.

This is a provisional file, not the final typeset article
